# Supplementary material for: Development and validation of a nomogram for assessment postoperative sodium disturbance in PAs patients: a retrospective cohort study
Source: PeerJ. 2023 Aug 30;11:e15946. doi: 10.7717/peerj.15946 (PMC10474829; doi:10.7717/peerj.15946)
Supplement: Supplemental Information 1 [file peerj-11-15946-s001.docx]

| Supplement Table 1 Variables in different types of preoperative biochemistry indices | |
| --- | --- |
| Types | Variables |
| Coagulation routine | prothrombin time (PT, S) |
|  | prothrombin activity (PTA, %) |
|  | prothrombin time ration (PTR) |
|  | prothrombin time international normalized ratio (PTINR) |
|  | fibrinogen (g/L) |
|  | activated partial thromboplastin time (APTT, S) |
|  | thrombin time (TT, S) |
|  | D-dimer (mg/L) |
| Blood routine examination | high-sensitivity C-reaction protein (hs-CRP, mg/L) |
|  | C-reaction protein (CRP, mg/L) |
|  | serum amyloid A (SAA, mg/L) |
|  | white blood cells (WBC, 10^9/L) |
|  | red blood cell (RBC, 10^12/L) |
|  | hemoglobin (HGB, g/L) |
|  | platelet count (PLT, 10^9/L) |
|  | hematocrit (HCT) |
|  | mean red cell volume (MCV, fL) |
|  | mean corpuscular hemoglobin (MCH, pg) |
|  | mean corpuscular hemoglobin concentration (MCHC, g/L) |
|  | coefficient of variation of red blood cell distribution (RDW-CV) |
|  | standard deviation of red blood cell distribution (RDW-SD, fL) |
|  | lymphocyte percentage (LYM, %) |
|  | neutrophil percentage (NEUT, %) |
|  | percentage of monocyte (MONO, %) |
|  | eosinophils percentage (EOS, %) |
|  | basophils percentage (BASO, %) |
|  | lymphocyte (LYM, 10^9/L) |
|  | neutrophils (NEUT, 10^9/L) |
|  | monocytes (MONO, 10^9/L) |
|  | eosinophils (EOS, 10^9/L) |
|  | basophils (BASO, 10^9/L) |
|  | plateletocrit (PCT, %) |
|  | mean platelet volume (MPV, fL) |
|  | platelet distribution width (PDW, fL) |
|  | platelet large cell ratio (P-LCR, %) |
|  | percentage of circulating reticulocytes (RET, %) |
|  | reticulocyte (RET, 10^9/L) |
|  | immature reticulocyte fraction (IRF, %) |
| routine biochemistry | alanine aminotransferase (ALT, U/L) |
|  | aspartate aminotransferase (AST, U/L) |
|  | total bilirubin (TBIL, umol/L) |
|  | direct bilirubin (DBIL, umol/L) |
|  | indirect bilirubin (IBIL, umol/L) |
|  | ϒ-glutamyltransferases (GGT, U/L) |
|  | alkaline phosphatase (ALP, U/L) |
|  | serum potassium (K, mmol/L) |
|  | serum sodium (Na, mmol/L) |
|  | serum chlorine (CL, mmol/L) |
|  | serum calcium (Ga, mmol/L) |
|  | serum phosphorus (P, mmol/L) |
|  | serum magnesium (Mg, mmol/L) |
|  | cystatin C (CysC, mg/L) |
|  | urea (mmol/L) |
|  | creatinine (umol/L) |
|  | urea nitrogen/creatinine (Bun/Cr, mg/mg) |
|  | capable of binding carbon dioxide (CO2-CP, mmol/L) |
|  | uric acid (UA, umol/L) |
|  | glucose (mmol/L) |
|  | β-hydroxybutyric acid (HBUT, mmol/L) |
|  | total cholesterol (T-Chol, mmol/L) |
|  | low-density lipoprotein-cholesterol (LDL-C, mmol/L) |
|  | high-density lipoprotein-cholesterol (HDL-C, mmol/L) |
|  | triglycerides (TG, mmol/L) |
|  | apolipoprotein A1 (ApoA1, g/L) |
|  | apolipoprotein B (ApoB, g/L) |
|  | apolipoprotein E (ApoE, g/L) |
|  | prealbumin (PA, g/L) |
|  | total protein (TP, g/L) |
|  | albumin (ALB, g/L) |
|  | globulin (GLB, g/L) |
|  | albumin/globulin (A/G) |
|  | total bile acid (TBA, umol/L) |
|  | glycocholic acid (CG, mg/L) |
|  | phosphocreatine kinase (CK, U/L) |
|  | lactate dehydrogenase (LDH, U/L) |
|  | creatine kinase lsoenzyme (CK-MB, U/L) |
|  | cholinesterase (CHE, U/L) |
|  | leucyl aminopepidase (LAP, U/L) |
|  | retinol binding protein (RBP, mg/L) |
|  | ɑ-L-fucosidase (AFU, U/L) |
|  | lipase (LIP, U/L) |
|  | serum amylase (SAMY, U/L) |
|  | serum iron (Fe, umol/L) |
|  | unsaturated iron binding capacity (UIBC, umol/L) |
|  | total iron binding capacity (TIBC, umol/L) |
|  | serum iron saturation (Fe/TIBC, %) |
|  | serum ferritin (SF, ug/L) |
|  | transferrin (Tf, g/L) |
|  | adenosine dehydrogenase (ADA, U/L) |
|  | superoxide dismutase (SOD, U/ml) |
|  | free fatty acids (NEFA, umol/L) |
