# Supplementary material for: Development and validation of a nomogram for assessment postoperative sodium disturbance in PAs patients: a retrospective cohort study
Source: PeerJ. 2023 Aug 30;11:e15946. doi: 10.7717/peerj.15946 (PMC10474829; doi:10.7717/peerj.15946)
Supplement: Supplemental Information 2 [file peerj-11-15946-s002.docx]

| Supplement Table 2 Patients with missing data in each of the variables | | |
| --- | --- | --- |
| Variables | Number of missing | Percentage of missing |
| pt | 2 | 0.009615385 |
| pta | 2 | 0.009615385 |
| ptr | 2 | 0.009615385 |
| ptnir | 2 | 0.009615385 |
| fbg | 2 | 0.009615385 |
| aptt | 2 | 0.009615385 |
| tt | 2 | 0.009615385 |
| dd | 2 | 0.009615385 |
| alt | 4 | 0.019230769 |
| ast | 4 | 0.019230769 |
| Total bilirubin | 4 | 0.019230769 |
| Direct bilirubin | 11 | 0.052884615 |
| Indirect bilirubin | 12 | 0.057692308 |
| ggt | 4 | 0.019230769 |
| alp | 4 | 0.019230769 |
| k | 2 | 0.009615385 |
| na | 2 | 0.009615385 |
| cl | 2 | 0.009615385 |
| ga | 2 | 0.009615385 |
| p | 11 | 0.052884615 |
| mg | 12 | 0.057692308 |
| cysc | 21 | 0.100961538 |
| Urea | 2 | 0.009615385 |
| Creac | 2 | 0.009615385 |
| Uric Acid Nitrogen and Creatinine | 144 | 0.692307692 |
| Carbon dioxide binding capacity | 3 | 0.014423077 |
| ua | 12 | 0.057692308 |
| glu | 2 | 0.009615385 |
| Hydroxybutyric acid | 13 | 0.0625 |
| chol | 12 | 0.057692308 |
| tg | 12 | 0.057692308 |
| hdlc | 12 | 0.057692308 |
| ldlc | 12 | 0.057692308 |
| Apolipoprotein a | 12 | 0.057692308 |
| Apolipoprotein b | 12 | 0.057692308 |
| Apolipoprotein e | 67 | 0.322115385 |
| pa | 12 | 0.057692308 |
| tp | 5 | 0.024038462 |
| ALB | 5 | 0.024038462 |
| glb | 5 | 0.024038462 |
| Albumin globulin | 12 | 0.057692308 |
| tba | 12 | 0.057692308 |
| cg | 128 | 0.615384615 |
| ck | 10 | 0.048076923 |
| ldh | 10 | 0.048076923 |
| ckmb | 10 | 0.048076923 |
| c-reactive protein | 11 | 0.052884615 |
| ceh | 6 | 0.028846154 |
| lap | 45 | 0.216346154 |
| rbp | 68 | 0.326923077 |
| afu | 12 | 0.057692308 |
| lip | 13 | 0.0625 |
| samy | 12 | 0.057692308 |
| Fe | 12 | 0.057692308 |
| uibc | 12 | 0.057692308 |
| tibc | 12 | 0.057692308 |
| Iron saturation | 146 | 0.701923077 |
| sf | 130 | 0.625 |
| tf | 12 | 0.057692308 |
| ada | 13 | 0.0625 |
| sod | 12 | 0.057692308 |
| nefa | 13 | 0.0625 |
| wbc | 0 | 0 |
| rbc | 0 | 0 |
| hgb | 0 | 0 |
| plt | 3 | 0.014423077 |
| hct | 2 | 0.009615385 |
| mcv | 2 | 0.009615385 |
| mch | 2 | 0.009615385 |
| mchc | 2 | 0.009615385 |
| rdwcv | 2 | 0.009615385 |
| rdwsd | 2 | 0.009615385 |
| lym | 2 | 0.009615385 |
| neut | 2 | 0.009615385 |
| mono | 2 | 0.009615385 |
| eos | 2 | 0.009615385 |
| baso | 2 | 0.009615385 |
| lyw | 2 | 0.009615385 |
| neutnum | 2 | 0.009615385 |
| mononum | 2 | 0.009615385 |
| eosnum | 2 | 0.009615385 |
| basonum | 2 | 0.009615385 |
| pct | 2 | 0.009615385 |
| mpv | 2 | 0.009615385 |
| pdw | 2 | 0.009615385 |
| plcr | 0 | 0 |
| ret | 0 | 0 |
| retnum | 0 | 0 |
| irf | 0 | 0 |
| sodium disturbance | 0 | 0 |
